# Early visual quality outcomes after small-incision lenticule extraction surgery for correcting high myopic astigmatism

**DOI:** 10.1186/s12886-021-01807-8

**Published:** 2021-01-19

**Authors:** Xiangtao Hou, Kaixuan Du, Dan Wen, Shengfa Hu, Tu Hu, Chenling Li, Yanhui Tang, Xiaoying Wu

**Affiliations:** 1 Eye Center of Xiangya Hospital, Central South University, Changsha, 410008 Hunan China; 2 Hunan Key Laboratory of Ophthalmology, Central South University, Changsha, 410008 Hunan China

## Abstract

**Background:**

To evaluate early optical quality outcomes after small-incision lenticule extraction (SMILE) surgery for correcting high myopic astigmatism.

**Methods:**

This retrospective study enrolled 55 eyes from 37 patients who had preoperative myopic astigmatism of ≥2.00 diopters (D) who had been treated with SMILE surgery. Preoperatively, the mean cylinder was − 2.41 ± 0.54 D (range, − 2.00 D to − 4.50 D). The preoperative and postoperative visual outcomes, refraction, and higher-order aberration (HOA) at 1 and 3 months were compared. Refractive astigmatism changes were analyzed by the Alpins vector method.

**Results:**

Three months after SMILE surgery, the average cylinder was − 0.14 ± 0.31 D, and the average astigmatism vector was − 0.09 D × 6.34°. The angle of error (AofE) was limited to within ±10°, and the magnitude of error was limited to within ±1.0 D in all patients. The correction index (CI) was 0.98 ± 0.07, the index of success (IOS) was 0.08 ± 0.13, and the flattening index (FI) was 0.97 ± 0.07. Significant positive correlations were found between IOS and |AofE| (*P* = 0.000); negative correlations were found between FI and |AofE| (*P* = 0.000). The postoperative total HOA, spherical aberration, vertical coma aberration, and trefoil 30° were increased significantly compared with preoperative measurements, and the increase in HOA was closely related to preoperative astigmatism (*P* < 0.05).

**Conclusions:**

SMILE has preferable outcomes for correcting high myopic astigmatism. Axis rotation during the surgery might influence the undercorrection of astigmatism. The increase of HOA after surgery is related to preoperative astigmatism.

## Background

Small-incision lenticule extraction (SMILE) is a newly developed surgical technique where the intrastromal lenticule is removed through a small arcuate incision for correcting refractive errors. Many existing studies have demonstrated the safety profiles and promising visual and refractive outcomes of SMILE [1–3].

Several concerns have been raised regarding its capability for correcting astigmatism, given the lack of cyclotorsion control on the VisuMax femtosecond laser used and the completely surgeon-dependent centration [4–6]. However, a few studies have proven that SMILE surgery can correct myopic astigmatism safely and effectively even in the presence of high astigmatism [7, 8]. Hence, we examined in the present study the safety, effectiveness, and predictability of SMILE for high myopic astigmatism, axis rotation during surgery, and improvement in visual quality, and then analyzed the possible influencing factors.

## Methods

### Study design and patients

This retrospective study involved 37 patients (55 eyes) who underwent SMILE surgery for correcting myopia and myopic astigmatism between April 2017 and May 2019 at Xiangya Hospital, Central South University, China. The inclusion criteria were: minimum age of 18 years, myopia (sphere measurement of < 10.00  diopters [D]), myopia astigmatism (cylinder measurement of ≥2.00 D), stable refractive error (refractive error change of ≤0.50 D in the past 2 years), clear cornea without opacity, central corneal thickness of > 460 μm, calculated residual stroma of > 280 μm, minimum 3 months’ follow-up, and no other pathologic ocular conditions except refractive error. The patients were instructed to stop wearing spherical contact lenses for at least 1 or 2 weeks, cylindrical and rigid contact lenses for at least 3–4 weeks, and orthokeratology lenses for at least 12 weeks [9]. This study adhered to the tenets of the Declaration of Helsinki and was approved by the Xiangya Hospital Ethics Committee.

### Observation criteria before and after surgery

The preoperative assessments included complete medical and ophthalmological history and comprehensive ophthalmic examination. The observation criteria before surgery and 1 and 3 months post-surgery included visual acuity, diopter, intraocular pressure by non-contact tonometer (CT-80, Topcon, Tokyo, Japan), corneal thickness by A-scan ultrasound (UP-1000, NIDEK, Tokyo, Japan), objective optical quality by an optical quality analysis system (OQAS™ II, Visiometrics, Terrassa, Spain), and anterior corneal surface higher-order aberrations (HOA) and corneal topography by Pentacam (OCULUS GmbH, Wetzlar, Germany).

### Vector method for astigmatism

**Fig. 1:**
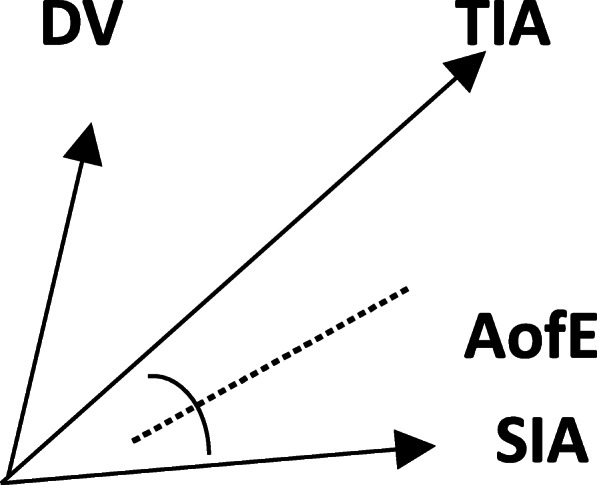
Schematic diagram of astigmatism vectors and their relationships. TIA: target induced astigmatism; SIA; surgically induced astigmatism; DV; difference vector; AofE:angle of error

Astigmatism correction was evaluated mainly based on the definitions and formulas by Alpins [10–12]. As suggested by Alpins (Fig. 1), target induced astigmatism (TIA) was defined as the astigmatic change the surgery was intended to induce. Here, the TIA was equal to the preoperative cylinder. The surgically induced astigmatism vector (SIA), defined as the surgery-induced astigmatic change, and the difference vector (DV) were equal to postoperative astigmatism. The angle of error (AofE) was the angle between the SIA and TIA vectors. The magnitude of error (MofE) was defined as the arithmetic difference between the magnitude of the TIA and SIA (MofE = |SIA| - |TIA|). The correction index (CI) was defined as the ratio of the magnitude of the SIA and TIA (CI = |SIA|/|TIA|). Preferably, the value should be 1, as a value of < 1 represents astigmatic undercorrection. The index of success (IOS) was defined as the ratio of the magnitude of postoperative astigmatism to the magnitude of the TIA (IOS = |DV|/|TIA|). Preferably, the value should be 0. The flattening index (FI) refers to the ratio of the corrected astigmatism to the expected corrected astigmatism in the direction of astigmatism expected to be corrected.

### Measurement of HOA

The root mean square (RMS) values of coma (Z_3_^− 1^, Z_3_^1^), spherical aberration (SA) (Z_4_^0^), trefoil (Z_3_^− 3^, Z_3_^3^), and total HOA (t-HOA, third- to sixth-order aberrations) were analyzed for 4 mm pupil diameter by the Pentacam.

### Surgical technique

All SMILE procedures were performed under surface anesthesia by a single experienced surgeon using a VisuMax femtosecond laser system (Carl Zeiss Meditec AG, Jena, Germany) with an established technique involving a repetition rate of 500 kHz and pulse energy of 140 nJ. The sphere was overcorrected by approximately 10%, and the cylinder was precisely corrected according to our experience to achieve emmetropia. The parameters used for all cases were: the cap diameter was set to 7.0–7.6 mm; the cap thickness was 110–130 μm; the lenticule diameter was 5.9–6.5 mm; and incision width of 2 mm at a position at 120°; the side cut angle was 90°. During the operation, the lens center was positioned as the watermark center. Following the cutting procedure, the lenticule was separated and removed from the side cut incision.

### Preoperative and postoperative care and follow-up

Each patient was prescribed levofloxacin eyedrops 3 days preoperatively. Patients with dry eye could add sodium hyaluronate eyedrops. Postoperatively, all patients received hormone eye drops (tobramycin dexamethasone eyedrops four times a day for the first week, Flutamide eyedrops three times a day for the second week, two times a day for the third week, and then withdraw). All patients were asked to follow up at 1 and 2 days, and 1 and 3 months after the operation.

### Statistical analysis

Statistical analysis was performed with SPSS 23.0 (IBM Corporation, Armonk, NY, USA). Normality was tested using the Kolmogorov–Smirnov test. Data that conformed to a normal distribution are reported as the means ± standard deviations; data that did not conform to the normal distribution are reported as the medians. A paired *t*-test and Wilcoxon test were used for preoperative and postoperative comparison. The preoperative and postoperative data and the influencing factors were analyzed using generalized estimation equations. All statistical tests were performed with a 95% confidence level (*P* < 0.05).

## Results

### Basic information of patients

Table 1 summarizes the basic information of all patients included in this study. SMILE surgery was successful for correcting myopia and myopia astigmatism in all eyes, and all surgeries were completed without intraoperative or postoperative complications.

**Table 1:** Preoperative demographics of patients with astigmatism

Characteristic	Value
No. of patients	37
No. of eyes	55
Sex (M/F)	15/22
Age (y)	22 (range, 17–40)
Manifest spherical equivalent (D)	−6.48 ± 1.46 (range from − 3.63 to − 9.88)
Manifest refractive cylinder (D)	−2.41 ± 0.54 (range from − 2.00 to −4.50)
UCVA (logMAR)	1.10
BCVA (logMAR)	− 0.18
Intraocular pressure (mmHg)	15

*D* Diopter, *UCVA* Uncorrected visual acuity, *BCVA* Best corrected visual acuity

### Effectiveness

**Fig. 2:**
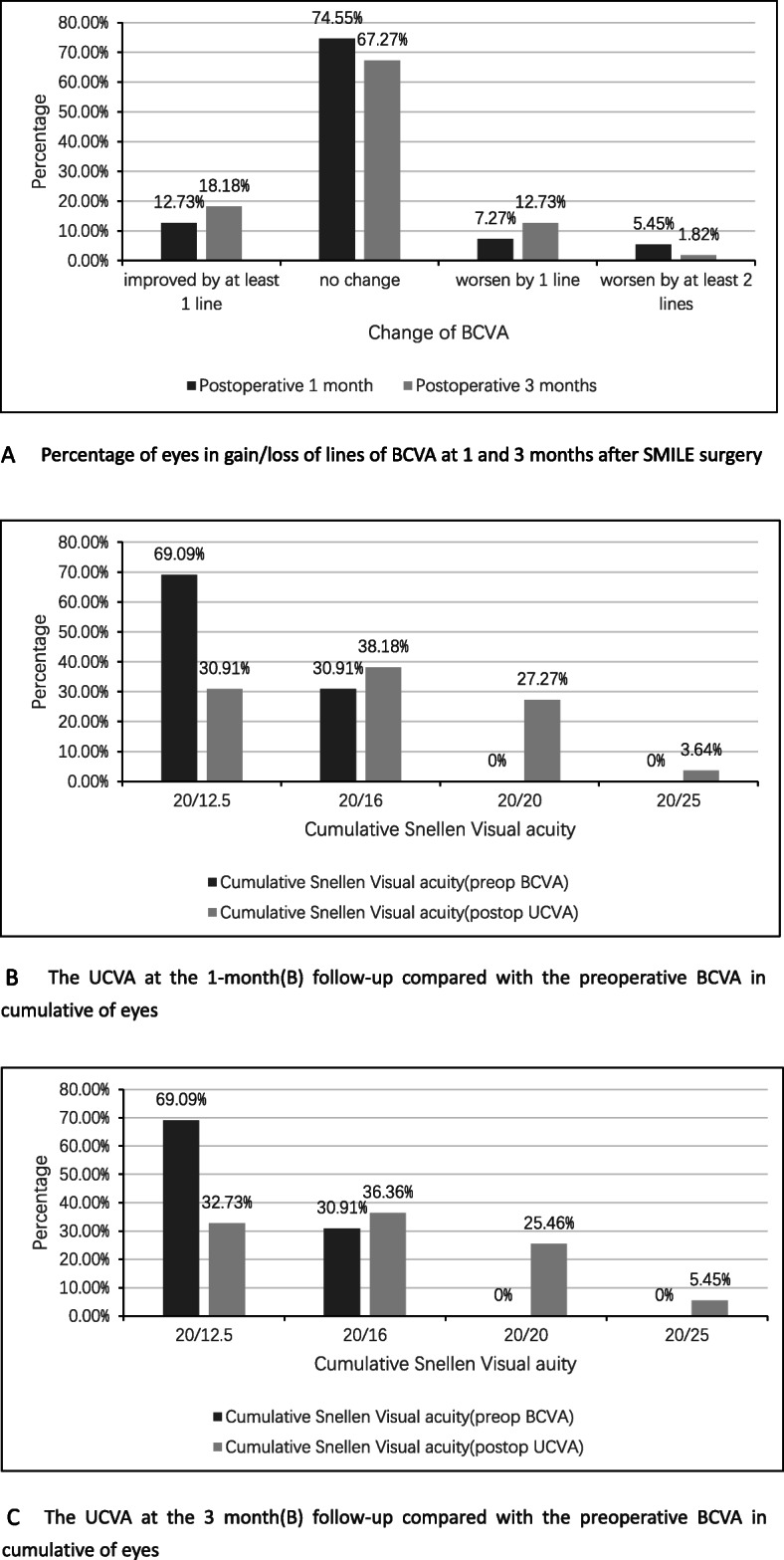
The UCVA at follow-up compared with the preoperative BCVA in cumulative of eyes and change in lines of BCVA. **a** Percentage of eyes in gain/loss of lines of BCVA at 1 and 3 months after SMILE surgery. **b**, **c** The UCVA at the 1-month (**b**) and 3-month (**c**) follow-up compared with the preoperative BCVA in cumulative eyes

Following SMILE, uncorrected visual acuity (UCVA) improvement occurred postoperatively in all enrolled patients (Table 2). There were significant differences in logMAR (logarithm of the minimum angle of resolution) UCVA between the 1-month and 3-month follow-up visits (*P* < 0.001). There were no significant differences in UCVA (*P* = 0.884), best corrected visual acuity (BCVA, *P* = 0.516), and the efficacy index (*P* = 0.690). Figure 2 shows the cumulative percentage of eyes that achieved definite cumulative levels of UCVA at 2 days and 1 and 3 months post-surgery. The median efficacy index at 2 days and 1 and 3 months post-surgery was 0.67, 0.80, and 0.83, respectively.

**Table 2:** Changes in visual acuity and diopter before and after SMILE surgery

Parameter	Preoperative	Postoperative		
		2 days	1 month	3 months
UCVA				
logMAR	1.10	0	− 0.08	− 0.08
≥ 20/25	0%	83.64%	98.18%	100%
≥ 20/20	0%	52.73%	83.64%	87.27%
BCVA				
logMAR	−0.18		−0.18	− 0.18
≥ 20/25	100%		100%	100%
≥ 20/20	100%		96.36%	98.18%
SE(D)	−6.48 ± 1.46		−0.25	−0.25
Cylinder(D)	−2.41 ± 0.54		0	0

*D* Diopter, *UCVA* Uncorrected distance visual acuity, *BCVA* Best corrected distance visual acuity

### Safety outcomes

The Wilcoxon test showed that the median safety index was 1 at 1 and 3 months post-surgery, and there was no significant difference between the two follow-up visits (*P* = 0.401). Figure 2 shows the UCVA at the 1-month and 3-month follow-ups as compared with the preoperative BCVA in cumulative eyes and change in lines of BCVA.

### Predictability

**Fig. 3:**
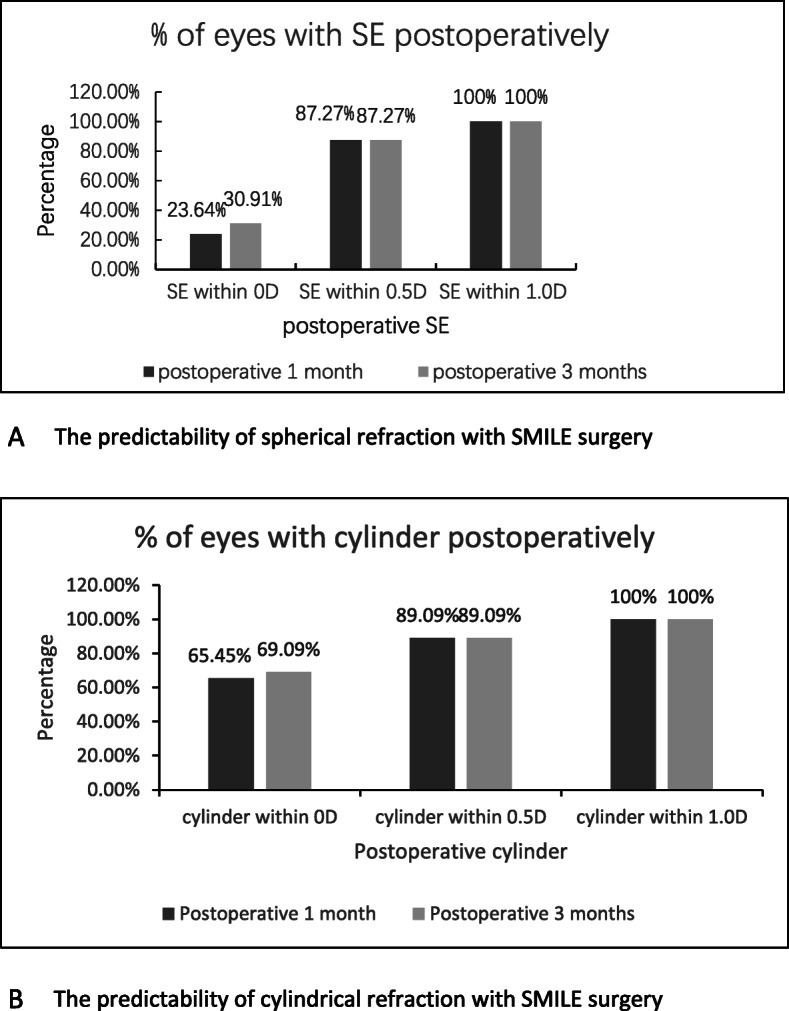
The predictability of SMILE surgery. **a** The predictability of spherical refraction with SMILE surgery. **b** The predictability of cylindrical refraction with SMILE surgery

The mean spherical equivalent (SE) was − 0.14 ± 0.35 D (range, − 0.75 D to + 0.50 D) and − 0.15 ± 0.36 D (range, − 0.75 D to + 0.50 D) at 1 and 3 months post-surgery, respectively, while the percentage of eyes with postoperative SE within ±0.5 D and ± 1.0 D were both 87.27 and 100% at 1 and 3 months (Fig. 3a).

**Fig. 4:**
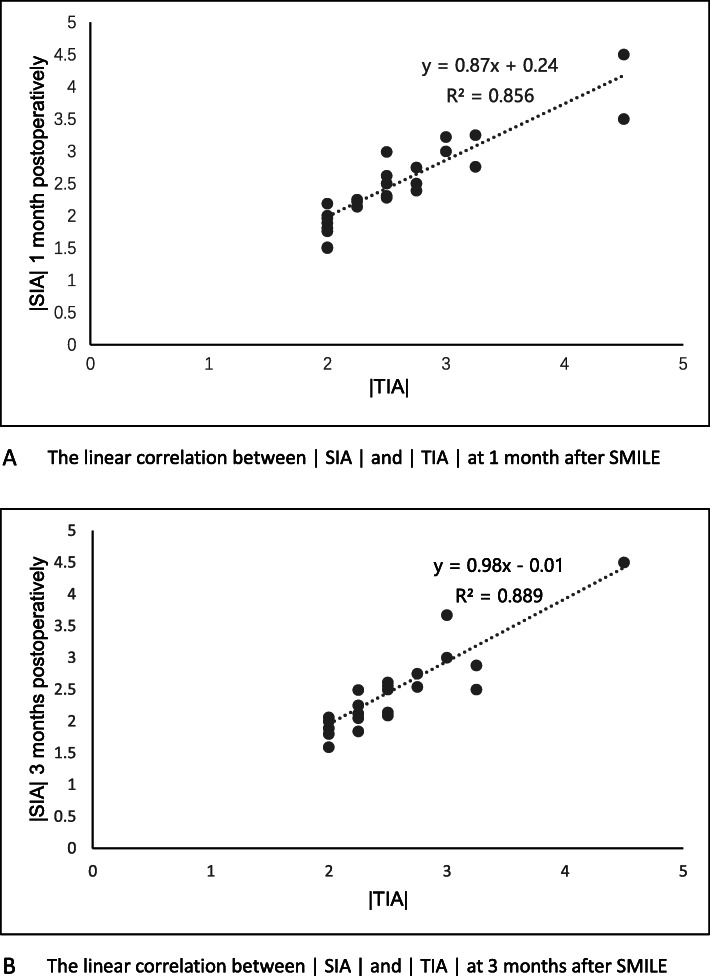
The linear correlation between the |SIA| and |TIA| after SMILE surgery. **a** The linear correlation between the |SIA| and |TIA| at 1 month post-surgery. **b** The linear correlation between the |SIA| and |TIA| at 3 months post-surgery

The mean cylinder was − 0.15 ± 0.33 D (range, − 1.00 D to + 0.50 D) and − 0.14 ± 0.31 D (range, − 1.00 D to + 0.75 D) at 1 and 3 months postoperatively, respectively; the percentage of eyes with postoperative cylinder within ±0.5 D and ± 1.0 D were both 89.09 and 100% at 1 and 3 months (Fig. 3b). There was a significant statistical association between |TIA| and |SIA| at 1 month and 3 months post-surgery (*r* = 0.947, Fig. 4a and 0.914, Fig. 4b, respectively).

### The vector method

**Fig. 5:**
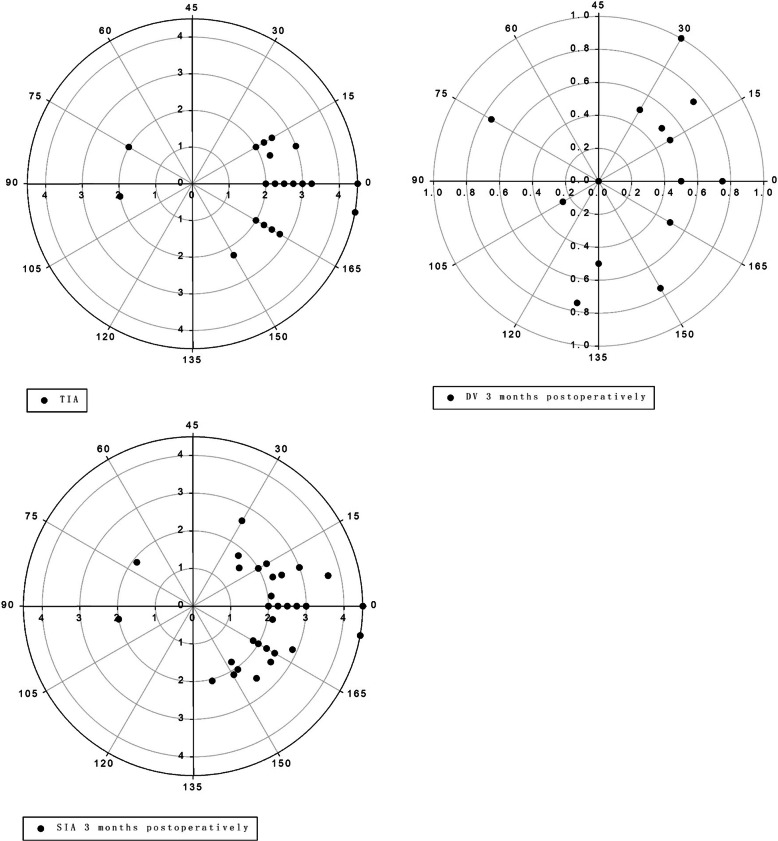
Double-angle figure of astigmatism 3 months after SMILE surgery

The double-angle plots demonstrate the TIA, SIA, and DV at the 3-month follow-up of 55 eyes (Fig. 5). The arithmetic mean TIA was 2.00–4.50 D in the small-incision lenticule. The TIA centroid coordinates were (x: 2.10 ± 0.99, y: − 0.26 ± 0.81), which indicated that the average astigmatism was with-the-rule before surgery. Postoperatively, the centroid coordinates of DV were (x: 0.08 ± 0.28, y: 0.07 ± 0.19) at 1 month, and (x: 0.09 ± 0.23, y: 0.01 ± 0.24) at 3 months, and the mean astigmatism in vector form was − 2.12 D × 7.06° preoperatively, − 0.11 D × 41.19° at 1 month post-surgery, and − 0.09 D × 6.34° at 3 months post-surgery. Table 3 shows the comparison of vector analysis between 1 and 3 months post-surgery.

**Table 3:** Comparison of vector analysis at 1 and 3 months after SMILE surgery

Parameter	Postoperative 1 month	Postoperative 3 months	*P*
SIA	2.35 ± 0.51	2.36 ± 0.57	0.819
DV	−0.15 ± 0.33	0.14 ± 0.31	0.919
CI	0.98 ± 0.07	0.98 ± 0.07	0.904
IOS	0.08 ± 0.12	0.08 ± 0.13	0.971
AofE	−1.16 ± 2.89°	−0.08 ± 3.56°	0.028*
|AofE|	1.29 ± 2.83°	1.81 ± 3.06°	0.278
MofE	−0.06 ± 0.20	−0.05 ± 0.19	0.819
FI	0.97 ± 0.07	0.97 ± 0.07	0.427

*CI* Correction index, *FI* Flattening index, *IOS* Index of success, *MofE* Magnitude of error, *SIA* Surgically induced astigmatism, *TIA* Target induced astigmatism

*P < 0.05 indicates significant difference

The Wilcoxon signed-rank test showed that there were no significant differences in the SIA (*P* = 0.819), DV (*P* = 0.919), CI (*P* = 0.904), IOS (*P* = 0.971), |AofE| (*P* = 0.278), MofE (*P* = 0.819), and FI (*P* = 0.427) between 1 month and 3 months post-surgery (Table 3).

The absolute AofE value deviated from the intended direction (Table 4). A positive value indicates a counterclockwise rotation from its intended axis, while a negative AofE value indicates a clockwise rotation. Table 4 shows that the AofE was significantly different between 1 and 3 months post-surgery: 45 eyes (81.82%) had |AofE| < 5°, while 10 eyes (18.18%) had |AofE| > 5° to ≤10°.

**Table 4:** Postoperative astigmatism at the 3-month follow-up of 55 eyes

Postoperative cylinder (D)	Absolute shift in axis (n)			
	≤5°	> 5°to≤10°	> 10°	total
0^a^	38	–	–	38
> 0.00 to≤ − 0.50	6	5	–	11
> 0.50 to≤ − 1.00	1	5	–	6
Total	45	10	–	55

Axis shift was determined from the postoperative to preoperative cylinder axis

^a^Shifts were determined as 0 for eyes with zero residual cylinder magnitude

**Fig. 6:**
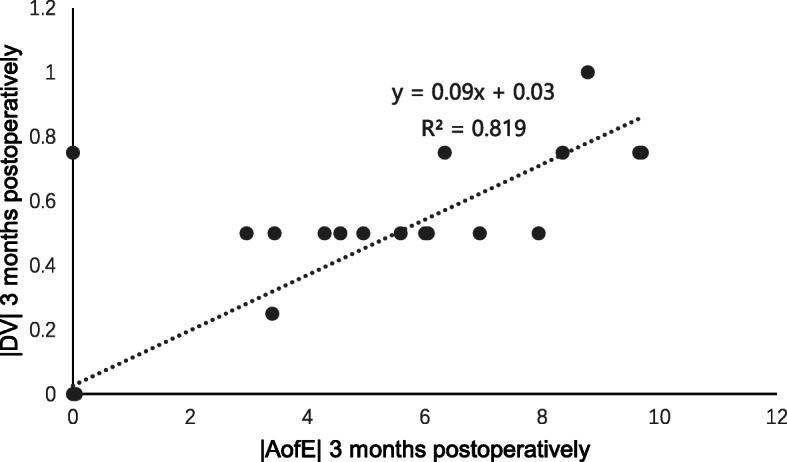
The linear correlation between the |DV| and |AofE| 3 months after SMILE surgery

**Fig. 7:**
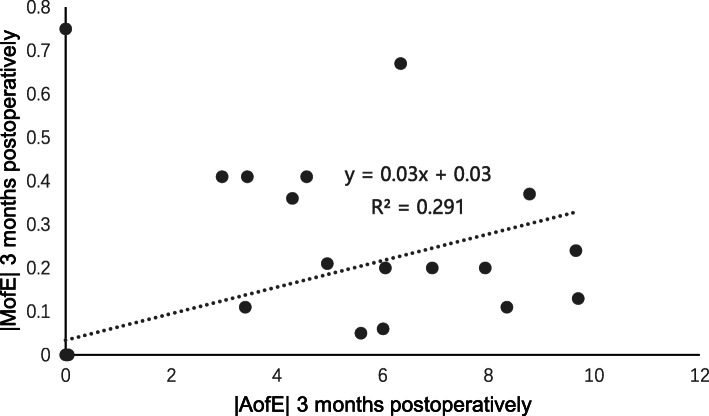
The linear correlation between the |AofE| and |MofE| 1 and 3 months after SMILE surgery

**Fig. 8:**
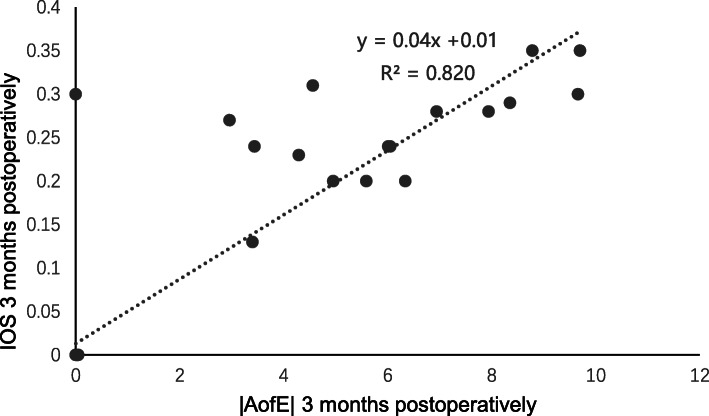
The linear correlation between the |IOS| and |AofE| at 3 months after SMILE surgery

**Fig. 9:**
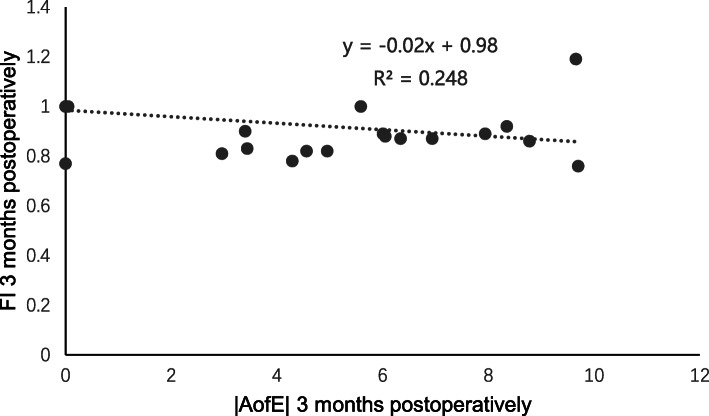
The linear correlation between the FI and |AofE| at 3 months after SMILE surgery

At 3 months post-surgery, the residual cylinder was − 0.14 ± 0.31 D (range, − 1.00 to + 0.75 D), the CI was 0.98 ± 0.07, the IOS was 0.08 ± 0.13, and the FI was 0.97 ± 0.07, which indicated slight undercorrection. Spearman correlation analysis at 3 months post-surgery showed a clear positive correlation between the |DV| and |AofE| (*r* = 0.737, *P* = 0.000, Fig. 6); a clear positive correlation was observed between the |MofE| and |AofE| (*r* = 0.694, *P* = 0.000, Fig. 7), which was the same as the relevance between the IOS and |AofE| (*r* = 0.699, *P* = 0.000, Fig. 8). Meanwhile, there was negative relevant relation between the FI and |AofE| (*r* = − 0.725, *P* = 0.000, Fig. 9), so there was a tendency toward undercorrection as the AofE between the corrected astigmatism and the target corrected astigmatism increased. However, there were no significant correlations between the |DV|, |MofE|, IOS, FI, CI, and |TIA| at 1 and 3 months post-surgery (*P* > 0.05), which indicated that the outcome of astigmatic correction mainly depends on the AofE instead of preoperative astigmatism.

The influence of binocular differences and repeated measurements were corrected using generalized estimation equations, and the influencing factors of UCVA and the absolute DV value were analyzed at 3 months post-surgery. The inclusion factors were: sex, age, preoperative intraocular pressure, preoperative spherical diopter, preoperative cylindrical diopter, preoperative astigmatic axis, preoperative anterior corneal surface curvature Km, intraoperative corneal cap thickness and diameter, lens thickness and diameter, and residual stromal thickness. The main factor affecting the logMAR UCVA at 3 months post-surgery was the preoperative astigmatic axis (*P* < 0.05). The influencing factors of the absolute DV value at 3 months post-surgery were: preoperative spherical diopter, preoperative cylindrical diopter, intraoperative lens thickness, lens diameter, and preoperative anterior corneal surface Km (*P* < 0.05).

### Corneal HOA before and after surgery

Table 5 shows the changes in the anterior corneal surface HOA before and after surgery. The t-HOA, spherical aberration, vertical coma aberration, and trefoil 30° increased significantly 3 months post-surgery compared with preoperative measurements (*P* < 0.05), while no significant difference was found for trefoil 0° and horizontal coma aberration before surgery or 1 and 3 months post-surgery (*P* > 0.05).

**Table 5:** HOA of the anterior corneal surfaces at 4 mm in diameter preoperatively and at 1 and 3 months postoperatively

Parameter	Preoperative	Postoperative	
		1 month	3 months
t-HOA RMS	0.11	0.23*	0.21*
Spherical aberration	0.03 ± 0.03	−0.07 ± 0.07*	−0.06 ± 0.07*
Vertical coma	−0.01 ± 0.07	−0.07 ± 0.10*	− 0.10 ± 0.09 *
Horizontal coma	−0.01 ± 0.05	− 0.01 ± 0.08	−0.00 ± 0.09
Trefoil 0°	0.00 ± 0.03	0.02 ± 0.07	0.02 ± 0.07
Trefoil 30°	−0.02 ± 0.04	0.01 ± 0.08**	0.03 ± 0.09*

*P < 0.001, **P < 0.01

*t-HOA RMS* Root mean square of total higher-order aberration

*P* < 0.05 indicates significant difference

Generalized estimation equations were performed between the anterior corneal surface HOA at 3 months post-surgery and the refractive diopter before surgery and 3 months after the surgery. The inclusion factors were: absolute preoperative spherical diopter value, absolute preoperative cylindrical diopter value, absolute spherical diopter value 3 months post-surgery, and absolute cylindrical diopter value 3 months post-surgery. The t-HOA at 3 months post-surgery correlated positively with the absolute values of the preoperative spherical diopter and cylindrical power (*P* < 0.05). The absolute values of the spherical aberration and the vertical coma at 3 months post-surgery correlated positively only with the preoperative cylindrical diopter (P < 0.05). This showed the t-HOA, spherical aberrations, and vertical coma aberrations increased at 3 months after the surgery as the preoperative astigmatism increased.

## Discussion

Previous studies have demonstrated that SMILE has shown excellent efficacy, probable safety, and predictability for correcting myopia and myopic astigmatism [13–16]. Here, we demonstrate that SMILE surgery is effective, safe, and predictable for myopia astigmatism of > 2.00 D. At 3 months post-surgery, 48 eyes (87.27%) had UCVA of 20/20 or better, and 48 eyes (87.27%) had SE within ±0.50 D. The postoperative SE and UCVA in our study are similar to recently published results [15–17].

Only a few studies have evaluated correction of high astigmatism after SMILE, especially in the vector method. Alpins vector analysis can comprehensively evaluate the outcomes of corneal refractive surgery for correcting myopic astigmatism by using the amount of astigmatism and the axial direction at the same time. Here, the vector analysis showed that the mean astigmatism in vector form was − 2.12 D × 7.06° preoperatively, − 0.11 D × 41.19° at 1 month post-surgery, and − 0.09 D × 6.34° at 3 months post-surgery. These results indicate a reduction in the cylinder value and that the axis of astigmatism was rotated at 1 month after the operation, which is considered to be related to early postoperative wound healing and the inflammatory response [18]. As the corneal healing response stabilized at 3 months, the axis of astigmatism mostly reversed, but the overall axis deviation of astigmatism was small.

Some studies have shown that there is a slight tendency toward undercorrection when treating astigmatism with SMILE [4, 8, 19, 20]. Ivarsen and Hjortdal [21] reported an undercorrection of 13% per diopter of attempted cylinder correction in low astigmatic eyes (< 2.5 D) and 16% per diopter in highly astigmatic eyes (≥2.5 D) after SMILE; they believed that the greater the preoperative astigmatism, the higher the degree of undercorrection. Pedersen et al. [19] reported that SMILE treatment of astigmatism seems to be predictable and effective, but with an astigmatic undercorrection of approximately 11%. Therefore, some researchers have suggested that the TIA should be increased by 10% based on the original cylindrical diopter before surgery when correcting astigmatism with SMILE [5]. Chan et al. [8] reported that, in eyes with high myopic astigmatism, SMILE offered the same astigmatic correction efficacy as LASIK (laser in situ keratomileusis). The authors mentioned that the perfect astigmatism treatment is attributed to strict center positioning during the operation and higher measurement accuracy of the preoperative cylindrical diopter. Our results indicate the desirable astigmatic correction (≥2.00 D). Postoperative astigmatism vector analysis demonstrated only a slight undercorrection. The main factor affecting the logMAR UCVA at 3 months post-surgery was the preoperative astigmatism axis (*P* < 0.05). The influencing factors of the absolute error vector value at 3 months post-surgery were preoperative spherical diopter, preoperative cylindrical diopter, intraoperative lens thickness, lens diameter, and preoperative anterior corneal surface Km (*P* < 0.05), which further suggests the importance of preoperative diopter measurement accuracy and strict central positioning during the operation. Also, our study indicates that the residual astigmatism axis in the early postoperative period turned clockwise from the expected correction, which is different from the results of Pedersen et al. [19], who reported that the astigmatism axis rotated counterclockwise. Chan et al. [22] observed a slight rotation of the cylinder axis (− 6.9°) in eyes with a temporal opening incision, although this was not statistically different from eyes with a superior incision (− 0.39°). In the present study, the position of the side incision in both eyes was set at 120°, and that in the study of Pedersen et al. [19] was 30–60°, which suggests that the slight axial rotation in the early postoperative period of SMILE might be related to the surgical incision location and the healing response of the corneal incision. However, the exact reasons remains to be studied further via increased sample sizes and different incision positions.

Previous research has shown that the effect of astigmatic correction is mostly affected by the magnitude and direction of astigmatism corrected during the operation, and the type and source of astigmatism, wound healing response, laser energy, cutting center positioning, and cutting depth might also account for this [18, 23–26]. In the present study, we show that the influencing factors of the absolute DV value at 3 months post-surgery are preoperative spherical diopter, preoperative cylindrical diopter, intraoperative lens thickness, lens diameter, and preoperative anterior corneal surface curvature Km (*P* < 0.05). Some studies have also reported that the position of the patient’s head, the rotation of their eyeballs during surgery, and the displacement of the pupil center might be the influencing factors that cause the axis rotation. As the body position changes, the eyeball rotates unconsciously, which would cause a deviation between the axis set before surgery and the axis corrected during surgery. If the eyeball were rotated > 2° without correction, it would not only affect the correction of astigmatism, but would also induce significant aberrations [27, 28]. When correcting astigmatism, inaccurate positioning of the astigmatism axis might cause undercorrection. An astigmatism axial deviation of 4°, 6°, 10°, 15°, and 30° would cause 14, 20, 35, 52%, and complete astigmatism undercorrection, respectively [29, 30]. In our study, the correlation analysis also showed that the absolute AofE value correlated positively with the absolute values of IOS and DV, and correlated negatively with the FI, indicating that accurate axial alignment and twist inspection are the key factors to achieving good visual quality after surgery. Ganesh et al. [31] observed that 86% of 81 highly astigmatic eyes demonstrated ≤5° cyclotorsion, and none of the eyes had ≥10° cyclotorsion; the mean magnitude was 5.5°. Based on this, they recommended manual compensation of cyclotorsion error during SMILE under the guidance of preoperative limbal markings, and observed improved results in the high-astigmatism subgroup (> 1.5 D). Then, they concluded that manual compensation of the eyeball rotation angle during surgery was effective for solving the problem of cutting deviation caused by the astigmatism axial position change caused by the patient’s eyeball rotation. However, it has also been reported [32] that the manual marking method itself could introduce inconsistency with range of 3.8–6.0°. Here, we did not use the manual compensation method of corneal marking for intraoperative rotation error in SMILE surgery for astigmatic correction. However, the surgeon paid great attention during the operation to the correct positioning of the patient’s posture and head position and strict watermark center positioning for correcting astigmatism. The postoperative error angle of our study is similar to that of Ganesh et al. [31]. How the accuracy of astigmatism correction in SMILE surgery can improved remains a worthwhile topic for further discussion.

For HOA, the t-HOA, spherical aberration, vertical coma aberration, and trefoil 30° all increased significantly 3 months postoperatively (*P* < 0.05), which is consistent with the research of Jin et al. [33] and Liu et al. [34]. Jin et al. [33] observed the results of 196 eyes and found that after SMILE, the t-HOA of the anterior corneal surface increased, the magnitude of the horizontal coma and spherical aberration were more obvious, and the change of aberration was correlated to preoperative SE. Liu et al. [34] revealed that the difference in lenticule center positioning during SMILE surgery was likely to influence the postoperative HOA changes. The VN (vertex normal center) would be a better choice of reference for the optic zone center for SMILE compared with the PC (pupil center) in HOA production. In our study, the correlation analysis showed that the t-HOA was increased at 3 months post-surgery.

SMILE was mainly positively related to the preoperative spherical diopter and astigmatism, suggesting that as the expected correction degree before surgery increases, so does the thickness of the lens that has to be removed, and more corneal tissue needs to be cut, with the corresponding change in the corneal surface morphology, which leads to an increase in total postoperative HOA. The increase in spherical aberration and vertical coma after surgery was mainly related to preoperative astigmatism, suggesting that patients with high astigmatism might be more likely to have poor visual quality after surgery. Therefore, the aspheric design of the SMILE operation and strict alignment during the operation are very important, and can reduce the introduction of spherical aberration and vertical coma to a certain extent [35, 36]. Besides, the introduction of postoperative HOA might also be related to corneal cell apoptosis, hyperplasia and healing reactions, and poor tear film stability in the early postoperative period [18, 37]. The different inclusion criteria and differences in pupil size and measuring instruments can lead to disparate results; furthermore, the proficiency of the surgeon and the setting of the surgical parameters would also affect the correlation between postoperative aberration and diopter. Therefore, many clinical studies are needed to further explore the correlation between the two.

There were a few limitations in this study. First, we included only 37 patients (55 eyes), and the follow-up duration was relatively short. A larger sample size and longer observation durations are needed in the future. Second, for bilaterally treated patients, although the two eyes of the same patient cannot be considered independent, the variance between eyes is usually less than that between subjects. Hence, the overall variance of a sample of measurements combined from both eyes was likely to be an underestimation of the true variance, resulting in increased risk of type 1 error.

## Conclusion

SMILE is a good choice for correcting myopia and myopic astigmatism. The vector analysis method can analyze the clinical effect of the correction of astigmatism by corneal refractive surgery objectively and accurately. There was a slight tendency for undercorrection in high myopic astigmatism, and the degree of undercorrection was not only related to the deviation of the correction degree, but also to the deviation of the correction angle. The increase of HOA on the anterior corneal surface after surgery was closely related to preoperative astigmatism. Our findings provide a valuable reference for surgeons who seek better postoperative visual quality: during surgery, they should position the center of the visual axis as accurately as possible when using SMILE to correct high myopic astigmatism, and adjust nomograms or use manual compensation for rotation errors.

## Data Availability

Available upon request from the first author; Dr. Xiangtao Hou.

## Abbreviations

SMILE: Small incision lenticule extraction
FS-LASIK: Femtosecond laser in situ keratomileusis
UCVA: Uncorrected visual acuity
BCVA: Best corrected visual acuity
SE: Spherical equivalent
t-HOA: Total higher-order aberration
TIA: Target induced astigmatism
SIA: Surgically induced astigmatism
DV: Difference vector
AofE: The angle of error
MofE: The magnitude of error
CI: The correction index
IOS: The index of success
FI: The flattening index
